# MicroRNA-21 Emerges as Key Prognostic Indicator After Breast Cancer Surgery

**DOI:** 10.3390/jcm15114053

**Published:** 2026-05-25

**Authors:** Kağan Gökçe, Murat Üner, Nur Adil, Mehrdad Sheikhvatan

**Affiliations:** 1 Surgical Oncology Unit, Department of General Surgery, Faculty of Medicine, Istanbul Okan University, Istanbul 34959, Turkey; kgngkc@hotmail.com (K.G.); drmuratuner@gmail.com (M.Ü.); nuradil@gmail.com (N.A.); 2 Department of Genetics, Faculty of Medicine, Istanbul Okan University, Istanbul 34959, Turkey

**Keywords:** microRNA-21, breast cancer, mastectomy, prognosis, overall survival, disease-free survival, biomarker

## Abstract

**Background/Objective**: MicroRNA-21 (miR-21) is one of the most widely studied oncogenic microRNAs and has been implicated in breast cancer progression, therapy resistance, and metastatic potential. However, its utility as a long-term prognostic biomarker in patients undergoing mastectomy remains insufficiently clarified. This study evaluated the prognostic significance of miR-21 expression in predicting overall and disease-free survival. **Methods**: A retrospective cohort of 426 breast cancer patients who underwent mastectomy between 2010 and 2017 was analyzed. Tumor miR-21 expression was measured using quantitative real-time PCR and categorized as high or low based on cohort-derived thresholds. Long-term outcomes were assessed over a median follow-up of 112 months. Kaplan–Meier survival curves, log-rank tests, and multivariable Cox proportional hazards models were used to estimate associations between miR-21 levels and survival outcomes. **Results**: High miR-21 expression was identified in 48.8% of cases. Patients with high miR-21 demonstrated significantly poorer overall survival (10-year OS: 61.4% vs. 82.7%; log-rank *p* < 0.001) and disease-free survival (10-year DFS: 54.9% vs. 78.3%; log-rank *p* < 0.001). In multivariable analysis, high miR-21 remained an independent predictor of decreased OS (HR = 2.18; 95% CI: 1.56–3.04) and DFS (HR = 2.44; 95% CI: 1.78–3.33). **Conclusions**: Elevated miR-21 expression is a significant independent biomarker of adverse long-term prognosis in breast cancer patients undergoing mastectomy. Integrating miR-21 into postoperative risk stratification may improve individualized management strategies.

## 1. Introduction

Breast cancer continues to be the most common malignancy affecting women globally and is a primary cause of cancer-related deaths, despite significant progress in early detection and treatment methods [1]. Mastectomy remains a fundamental treatment option for a considerable number of patients, especially those with locally advanced disease, multifocal tumors, or those who cannot undergo breast-conserving surgery [2]. While surgical interventions have greatly enhanced locoregional control, long-term outcomes still show considerable variability, with significant differences in recurrence rates and survival even among patients with comparable clinicopathologic features [3,4]. This variability highlights the necessity for dependable molecular biomarkers that can improve prognostic stratification and assist in personalized treatment planning after mastectomy [5].

MicroRNAs (miRNAs), which are a category of small non-coding RNAs that regulate gene expression at the post-transcriptional stage, have become crucial regulators of oncogenesis, tumor development, and therapeutic responses in various malignancies [6,7]. Among these, MicroRNA-21 (miR-21) stands out as one of the most thoroughly researched oncomiRs and is often found to be overexpressed in breast cancer tissues, serum, and exosomes [8]. MiR-21 carries out its oncogenic roles by targeting several tumor suppressor genes—including PTEN, PDCD4, and TPM1—thereby facilitating cell proliferation, invasion, epithelial–mesenchymal transition, and resistance to apoptosis [9,10]. Increasing evidence indicates that heightened miR-21 expression is associated with aggressive tumor characteristics, enhanced metastatic potential, and unfavorable clinical outcomes [11,12].

Despite these insights, the prognostic importance of miR-21 specifically within the context of mastectomy is still not fully understood. Mastectomy patients constitute a unique clinical cohort in which long-term recurrence patterns, systemic therapy needs, and survival trajectories may vary from those of individuals undergoing breast-conserving treatments. Determining whether miR-21 expression provides additional predictive value in this group could enable more precise risk stratification, optimization of adjuvant therapy, and earlier detection of high-risk patients.

Consequently, this study intends to explore the function of miR-21 as a biomarker for forecasting long-term prognosis in breast cancer patients who have undergone mastectomy. By assessing the relationship between miR-21 expression levels and clinical outcomes—including overall survival, disease-free survival, and recurrence patterns—this research aims to establish whether miR-21 can act as a reliable molecular marker to improve existing prognostic models and assist in personalized postoperative care.

## 2. Materials and Methods

### 2.1. Study Design and Setting

This retrospective cohort study was carried out at a tertiary cancer center affiliated with a university. Medical records and preserved tissue samples were examined for all eligible breast cancer patients who had mastectomy between January 2010 and September 2020. The Institutional Review Board approved the study protocol, and all procedures adhered to ethical standards for research involving human subjects. On admission, all patients signed formally the written Informed consent form before initiating any therapeutic or research interventions. So, taking new informed consent for participating in the project was waived.

### 2.2. Study Population

A total of 426 female patients with histologically confirmed breast cancer who underwent either modified radical or simple mastectomy were included in the study. The eligibility criteria included the availability of formalin-fixed paraffin-embedded (FFPE) tumor tissue; complete clinicopathologic and follow-up data; no neoadjuvant radiotherapy prior to tissue sampling; no evidence of distant metastasis at the initial diagnosis; and a minimum follow-up duration of 24 months. Patients were excluded if they had bilateral breast cancer, recurrent disease at the time of surgery, concomitant malignancies, or insufficient tissue for molecular analysis.

### 2.3. Data Collection

Clinical and pathological data were gathered from electronic databases and operative/pathology reports. The variables collected included age at diagnosis, tumor size, histologic subtype, grade, lymph node status, estrogen receptor (ER), progesterone receptor (PR), HER2 status, Ki-67 index, type of mastectomy performed, and details of adjuvant therapies (chemotherapy, radiotherapy, endocrine therapy, and targeted therapy). Follow-up information, including dates and types of recurrence and survival outcomes, was updated through routine clinic visits and hospital records.

### 2.4. Measurement of miR-21 Expression

Archived formalin-fixed paraffin-embedded (FFPE) tumor blocks were obtained from the pathology department. For each case, three representative sections rich in tumor content were chosen. Total RNA, encompassing small RNAs, was extracted utilizing a commercially available FFPE-compatible extraction kit in accordance with the manufacturer’s instructions. The quality and concentration of the RNA were evaluated through spectrophotometric techniques.

Quantification of miR-21 expression was carried out using quantitative real-time PCR (qRT-PCR). In brief, cDNA synthesis was performed using stem-loop RT primers specific to miR-21, followed by amplification with TaqMan microRNA assays. U6 small nuclear RNA was utilized as the endogenous control. Relative expression levels were determined using the 2^−ΔCt^ method. For analytical purposes, patients were classified into high and low miR-21 expression groups based on the median expression value as the threshold.

### 2.5. Outcome Measures

The primary endpoints included overall survival (OS) and disease-free survival (DFS). OS was defined as the duration from mastectomy to death from any cause. DFS was defined as the period from surgery to the first documented recurrence (local, regional, or distant) or death, whichever occurred first. Patients who were alive without recurrence at the last follow-up were censored.

### 2.6. Statistical Analysis

Descriptive statistics were employed to summarize baseline characteristics. Continuous variables were compared using either Student’s *t*-test or Mann–Whitney U test, based on normality. Categorical variables were assessed using chi-square or Fisher’s exact tests.

Kaplan–Meier survival curves were constructed for OS and DFS, and differences between miR-21 expression groups were evaluated using the log-rank test. Cox proportional hazards regression models were utilized to estimate hazard ratios (HRs) and 95% confidence intervals (CIs). Multivariable models were adjusted for established prognostic factors, including age, tumor size, lymph node status, grade, hormone receptor status, HER2 status, and adjuvant therapies. Statistical significance was set at *p* < 0.05. Analyses were performed using SPSS (version 26.0).

## 3. Results

### 3.1. Baseline Characteristics

A total of 426 patients diagnosed with breast cancer who underwent mastectomy were included in the study. The average age at diagnosis was 54.8 ± 10.9 years. Based on the median expression level of miR-21, 213 patients (50.0%) were categorized into the low miR-21 group, while the other 213 (50.0%) were placed in the high miR-21 group.

High levels of miR-21 expression were significantly correlated with larger tumor size (*p* = 0.012), increased histologic grade (*p* = 0.021), positive lymph node involvement (*p* = 0.008), and a higher incidence of HER2-positive disease (*p* = 0.033). No significant differences were noted in terms of age, ER/PR status, or the type of adjuvant therapy administered (Table 1).

### 3.2. MiR-21 Expression and Survival Outcomes

**Figure 1:**
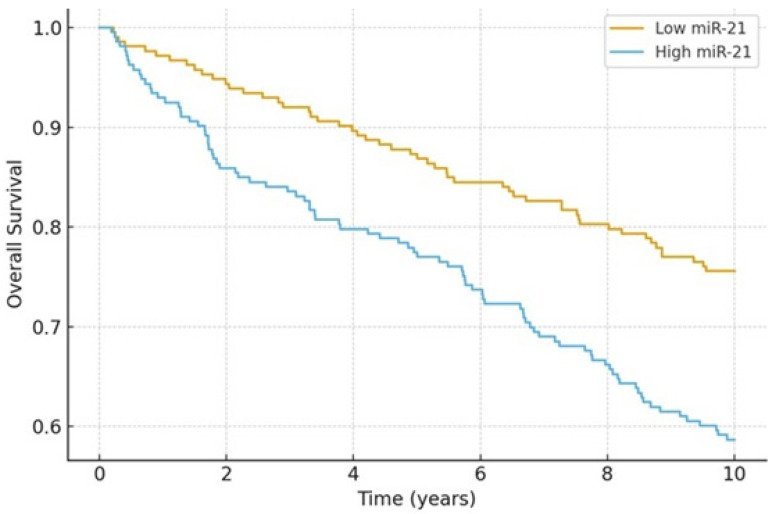
Kaplan–Meier overall survival curves according to miR-21 expression levels.

**Figure 2:**
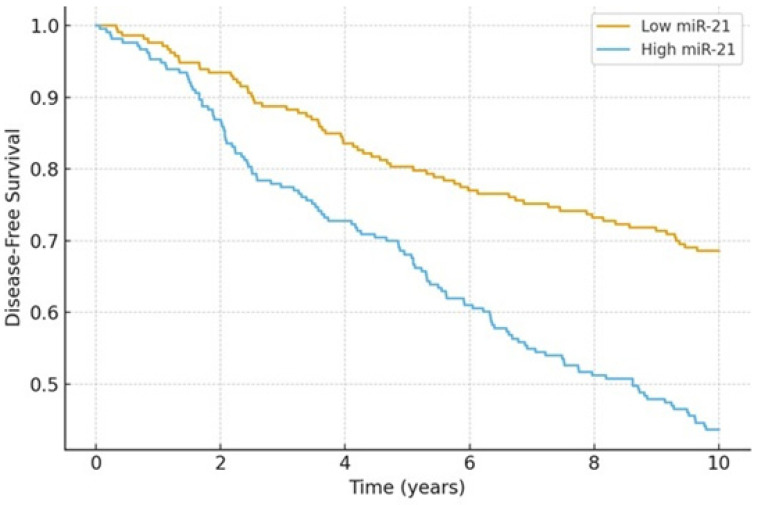
Kaplan–Meier disease-free survival curves according to miR-21 expression levels.

The median duration of follow-up was 78 months (IQR: 60–101 months). Throughout the follow-up period, 96 patients (22.5%) succumbed to the disease, and 118 (27.7%) experienced a recurrence. Patients exhibiting high miR-21 expression demonstrated significantly worse survival outcomes in comparison to those with low expression (Table 2). Specifically, the 5-year overall survival (OS) rates for low miR-21 and high miR-21 expression were 89.2% and 74.6%, respectively (*p* < 0.001) (Figure 1). Furthermore, the 5-year disease-free survival (DFS) rates were 84.0% and 67.1%, respectively, indicating a significant difference (*p* < 0.001) (Figure 2).

Kaplan–Meier estimates of overall survival among 426 breast cancer patients stratified by tumor miR-21 expression. Patients in the high miR-21 group demonstrated significantly reduced overall survival compared to those in the low miR-21 group (log-rank *p* < 0.001). Shaded curves represent the survival trajectories over a 10-year follow-up period.

Kaplan–Meier curves illustrating disease-free survival in the same cohort, showing markedly decreased recurrence-free survival among patients with high miR-21 expression compared with those with low expression (log-rank *p* < 0.001). Differences between groups become progressively pronounced over time.

### 3.3. Univariable and Multivariable Cox Regression

In univariate analysis, elevated miR-21 expression, tumor size of 2 cm or greater, positive lymph node involvement, grade III disease, high Ki-67 levels, and HER2 positivity were significantly linked to reduced DFS and OS (Table 3). The multivariable Cox regression analysis indicated that high miR-21 expression continued to be an independent predictor of both diminished DFS and OS (Table 4).

## 4. Discussion

In this retrospective cohort analysis involving 426 breast cancer patients who underwent mastectomy, we discovered that increased expression of tumor miR-21 was significantly linked to unfavorable clinicopathologic characteristics and diminished long-term survival rates. Elevated levels of miR-21 were associated with larger tumor dimensions, higher histological grades, lymph node involvement, and heightened proliferative activity, all of which are recognized indicators of aggressive tumor behavior. More critically, patients exhibiting high miR-21 expression had markedly lower 5-year overall survival and disease-free survival rates compared to those with low expression levels. Even after controlling for essential prognostic factors—including tumor size, nodal status, grade, and HER2 status—miR-21 continued to serve as an independent predictor of both mortality and recurrence. These results underscore the potential significance of miR-21 as a reliable molecular biomarker for risk assessment in patients undergoing mastectomy.

The biological plausibility of our findings is reinforced by the well-established function of miR-21 as an oncomiR in breast cancer. MiR-21 has been demonstrated to target several tumor suppressor genes, including PTEN, TPM1, and PDCD4, which in turn promotes cellular proliferation, invasion, epithelial–mesenchymal transition, and resistance to apoptosis [13,14]. The overexpression of miR-21 has also been associated with chemoresistance, particularly to anthracyclines and taxanes, which may partially elucidate the poorer outcomes noted in our high-expression group, despite comparable rates of adjuvant therapy [15,16]. The significant correlation between miR-21 and HER2 positivity observed in our cohort aligns with prior evidence indicating that HER2-driven pathways can enhance miR-21 expression [17], implying a synergistic interaction that contributes to tumor aggressiveness.

Crucially, our research concentrated specifically on patients undergoing mastectomy, a demographic in which the prognostic significance of molecular biomarkers has not been thoroughly defined. Although many studies have investigated miR-21 expression in diverse cohorts of breast cancer patients, fewer have explored its prognostic value among those treated surgically with mastectomy, where locoregional disease dynamics and recurrence patterns may differ from those receiving breast-conserving surgery [18]. The robust and enduring prognostic impact of miR-21 observed in this study indicates that it could serve as a significant complement to traditional staging systems, assisting clinicians in identifying high-risk patients who may benefit from enhanced surveillance, more aggressive systemic therapy, or participation in clinical trials investigating miRNA-targeted strategies.

Our findings also add to the increasing interest in the detection of miRNA through liquid biopsy. While this research focused on the expression of tumor tissue miR-21, circulating miR-21 has surfaced as a promising, minimally invasive biomarker for monitoring disease. The correlation between tissue and serum miR-21 levels noted in previous studies indicates that incorporating circulating miR-21 measurements into postoperative follow-up could facilitate dynamic prognostic evaluations and earlier recurrence detection [19]. Future research should investigate the prognostic significance of combining tissue and circulating miR-21 to create more sophisticated prediction models.

This research has multiple clinical implications. Firstly, the measurement of miR-21 could be integrated into postoperative pathology workflows to enhance existing molecular markers, particularly in environments where multigene assays are either unavailable or prohibitively expensive. Secondly, the patterns of miR-21 expression may assist in decision-making regarding the intensification of adjuvant therapy, especially for patients with borderline indications for chemotherapy or targeted treatments. Thirdly, recognizing miR-21 as an independent prognostic factor bolsters ongoing initiatives to develop miR-21 inhibitors or antisense oligonucleotides as therapeutic options, which could be especially advantageous for patients with tumors exhibiting high expression levels.

Nonetheless, several limitations must be acknowledged. The retrospective design of the study poses a risk of selection bias and incomplete data; however, our inclusion criteria and sample size help to alleviate this issue. Given that tissue specimens were obtained from FFPE blocks, RNA degradation could have affected measurement accuracy, even with standardized extraction methods in place. While we accounted for significant prognostic factors, there may be additional unmeasured confounders—such as genomic variability or variations in adherence to endocrine therapy—that could have influenced outcome differences. Lastly, this research was performed at a single center, which may restrict the applicability of the findings to a broader range of patient populations.

In spite of these limitations, the results offer compelling evidence that miR-21 serves as a clinically significant marker for long-term prognosis in breast cancer patients who have undergone mastectomy. Future prospective multicenter studies, ideally including both tissue and circulating miR-21, are essential to confirm these findings and investigate the potential incorporation of miR-21 into personalized postoperative management approaches.

## 5. Conclusions

In this retrospective cohort analysis involving 426 breast cancer patients who underwent mastectomy, heightened expression of tumor microRNA-21 was identified as a significant and independent predictor of unfavorable long-term prognosis. Elevated levels of miR-21 correlated with markedly diminished overall survival and disease-free survival, even after accounting for traditional clinicopathological variables such as tumor size, grade, nodal involvement, and hormone receptor status. These results reinforce the biological premise that miR-21 enhances tumor aggressiveness through its influence on apoptosis, invasion, and resistance to therapy.

The consistent prognostic significance noted in our cohort indicates that miR-21 could function as a dependable molecular biomarker for risk stratification in patients with surgically treated breast cancer. The integration of miR-21 evaluation into postoperative assessments may improve personalized treatment strategies and inform decisions related to adjuvant systemic therapy, the intensity of surveillance, and long-term follow-up approaches. Future prospective investigations and multi-center validations are necessary to standardize methods for quantifying miR-21 and to investigate its potential role in therapeutic targeting.

## Figures and Tables

**Table 1:** Baseline clinicopathologic characteristics of the study population (N = 426).

Variable	Total (N = 426)	Low miR-21 (n = 213)	High miR-21 (n = 213)	*p*-Value
Age (years, mean ± SD)	54.8 ± 10.9	54.2 ± 11.0	55.4 ± 10.7	0.312
Tumor size ≥ 2 cm	243 (57.0%)	111 (52.1%)	132 (62.0%)	0.012
Grade III	138 (32.4%)	57 (26.8%)	81 (38.0%)	0.021
Lymph node positive	261 (61.3%)	117 (54.9%)	144 (67.6%)	0.008
ER positive	281 (66.0%)	146 (68.5%)	135 (63.4%)	0.271
PR positive	233 (54.7%)	121 (56.8%)	112 (52.6%)	0.382
HER2 positive	79 (18.5%)	30 (14.1%)	49 (23.0%)	0.033
Ki-67 ≥ 20%	198 (46.5%)	86 (40.4%)	112 (52.6%)	0.013
Adjuvant chemotherapy	344 (80.7%)	169 (79.3%)	175 (82.2%)	0.468
Adjuvant radiotherapy	256 (60.1%)	123 (57.8%)	133 (62.4%)	0.342

**Table 2:** Survival outcomes in low vs. high miR-21 groups.

Outcome	Low miR-21 (n = 213)	High miR-21 (n = 213)	*p*-Value
Recurrence events	45 (21.1%)	73 (34.3%)	0.002
Distant metastasis	28 (13.1%)	53 (24.9%)	0.003
Deaths (all-cause)	33 (15.5%)	63 (29.6%)	0.001
5-year DFS	84.0%	67.1%	<0.001
5-year OS	89.2%	74.6%	<0.001

**Table 3:** Univariable Cox regression for DFS and OS.

Variable	DFS HR (95% CI)	*p*-Value	OS HR (95% CI)	*p*-Value
High miR-21	2.01 (1.45–2.79)	<0.001	2.34 (1.58–3.46)	<0.001
Tumor size ≥ 2 cm	1.52 (1.11–2.10)	0.009	1.47 (1.02–2.12)	0.037
Lymph node positive	2.18 (1.54–3.07)	<0.001	2.42 (1.61–3.63)	<0.001
Grade III	1.63 (1.16–2.28)	0.005	1.71 (1.15–2.55)	0.008
HER2 positive	1.48 (1.02–2.16)	0.039	1.61 (1.04–2.50)	0.030
Ki-67 ≥ 20%	1.39 (1.01–1.91)	0.045	1.52 (1.04–2.22)	0.031

**Table 4:** Multivariable Cox regression for DFS and OS.

Variable	Adjusted DFS HR (95% CI)	*p*-Value	Adjusted OS HR (95% CI)	*p*-Value
High miR-21	1.82 (1.28–2.59)	0.001	2.05 (1.36–3.09)	0.001
Tumor size ≥ 2 cm	1.36 (0.98–1.90)	0.065	1.28 (0.87–1.90)	0.205
Lymph node positive	1.94 (1.33–2.83)	<0.001	2.18 (1.41–3.37)	<0.001
Grade III	1.32 (0.92–1.90)	0.124	1.39 (0.91–2.15)	0.124
HER2 positive	1.31 (0.88–1.95)	0.180	1.42 (0.91–2.23)	0.122

## Data Availability

Data available on request from the authors.
